# Multifunctional nanobubbles carrying indocyanine green and paclitaxel for molecular imaging and the treatment of prostate cancer

**DOI:** 10.1186/s12951-020-00650-1

**Published:** 2020-09-03

**Authors:** Minmin Lan, Lianhua Zhu, Yixuan Wang, Daijia Shen, Kejing Fang, Yu Liu, Yanli Peng, Bin Qiao, Yanli Guo

**Affiliations:** 1 Department of Ultrasound, Southwest Hospital, Army Medical University, No. 30 Gaotanyan Street, Shapingba District, Chongqing, 400038 China; 2 State Key Laboratory Of Silkworm Genome Biology, Southwest University, Beibei District, Chongqing, China; 3 Chongqing Medical University, Chongqing, China

**Keywords:** Nanobubble, Indocyanine green, Paclitaxel, Ultrasound molecular imaging, Photoacoustic imaging, Prostate cancer

## Abstract

**Background:**

Combining ultrasound imaging with photoacoustic imaging provides tissue imaging with high contrast and resolution, thereby enabling rapid, direct measurements and the tracking of tumour growth and metastasis. Moreover, ultrasound-targeted nanobubble destruction (UTND) provides an effective way to deliver drugs, effectively increasing the content of the drug in the tumour area and reducing potential side effects, thereby successfully contributing to the treatment of tumours.

**Results:**

In this study, we prepared multifunctional nanobubbles (NBs) carrying indocyanine green (ICG) and paclitaxel (PTX) (ICG-PTX NBs) and studied their applications in ultrasound imaging of prostate cancer as well as their therapeutic effects on prostate cancer when combined with UTND. ICG-PTX NBs were prepared by the mechanical oscillation method. The particle size and zeta potential of the ICG-PTX NBs were 469.5 ± 32.87 nm and − 21.70 ± 1.22 mV, respectively. The encapsulation efficiency and drug loading efficiency of ICG were 68% and 2.52%, respectively. In vitro imaging experiments showed that ICG-PTX NBs were highly amenable to multimodal imaging, including ultrasound, photoacoustic and fluorescence imaging, and the imaging effect was positively correlated with their concentration. The imaging effects of tumour xenografts also indicated that ICG-PTX NBs were of good use for multimodal imaging. In experiments testing the growth of PC-3 cells in vitro and tumour xenografts in vivo, the ICG-PTX NBs + US group showed more significant inhibition of cell proliferation and the promotion of cell apoptosis compared to the other groups (P < 0.05). Blood biochemical analysis of the six groups showed that the levels of aspartate aminotransferase (AST), phenylalanine aminotransferase (ALT), serum creatinine (CRE) and blood urea nitrogen (BUN) in the ICG-PTX NBs and the ICG-PTX NBs + US groups were significantly lower than those in the PTX group (P < 0.05). Moreover, H&E staining of tissue sections from vital organs showed no obvious abnormalities in the ICG-PTX NBs and the ICG-PTX NBs + US groups.

**Conclusions:**

ICG-PTX NBs can be used as a non-invasive, pro-apoptotic contrast agent that can achieve multimodal imaging, including ultrasound, fluorescence and photoacoustic imaging, and can succeed in the local treatment of prostate cancer providing a potential novel method for integrated research on prostate cancer diagnosis and treatment.

## Introduction

Prostate cancer is one of the most common malignancies of the male genitourinary system. In recent years, the incidence and mortality of prostate cancer in China has been increasing [1]. Early diagnosis and effective treatment are the basis for ensuring a good prognosis in patients with prostate cancer [2, 3]. Previous studies have shown that ultrasound molecular imaging provides new ideas for early diagnosis, treatment and efficient monitoring of prostate cancer due to its real-time dynamic imaging, therapeutic controllability and easy operation [4, 5]. However, due to the poor sensitivity and specificity of conventional ultrasound in the diagnosis of prostate cancer, the development of an ultrasound contrast agent with good sensitivity and strong contrast is urgent for current research in prostate cancer ultrasound molecular imaging [6]. Near-infrared fluorescence imaging has the advantages of real-time imaging, high sensitivity, no ionizing radiation use and reduced cost. It has been extensively used for the early diagnosis and targeted therapy of diseases; however, due to its strong optical scattering and limited penetration depth, which leads to poor spatial positioning, it has significant limitations in clinical applications [7]. Combining the high penetration depth of pure ultrasound imaging with the high contrast of pure optical imaging, photoacoustic imaging provides tissue imaging with high contrast and resolution, thereby enabling rapid, direct measurements and the tracking of tumour growth and metastasis, as well as real-time imaging and analysis of the obtained data. It has great application potential in tumour diagnosis and treatment monitoring [8]. Therefore, constructing a contrast agent suitable for the multimodal imaging of tumours such as ultrasound, fluorescence and photoacoustic imaging can overcome the limitations of a single imaging technique and is garnering attention.

Indocyanine green (ICG) is a near-infrared (NIR) fluorescent dye that has been approved by the US Food and Drug Administration (FDA). Current clinical practice shows that ICG can not only be used as a fluorescence probe for retinal angiography and cardiovascular and hepatic imaging but also for the accurate localization of certain carcinomas in situ and metastatic tumours. However, the shortcomings of ICG when used by itself in photoacoustic imaging, such as rapid clearance, poor stability, and lack of distribution specificity, form major obstacles in the diagnosis of tumours [9–13]. Certain studies have proposed the combination of ICG with carriers in order to prolong their circulation half-life and increase their efficacy. In this regard, studies have confirmed that liposomes have an appreciable affinity so that they can effectively encase ICG, improve its stability in circulation, and enhance the photoacoustic and fluorescent imaging of tumour tissues [14–17].

One of the current research directions of cancer treatment is the construction of a multifunctional nanodrug that can simultaneously integrate diagnosis and treatment. Multifunctional nanodrugs are targeted to diseased tissues by integrating therapeutic and imaging agents into nanoparticles, which can significantly improve the therapeutic effects and reduce toxic side effects on normal tissues [18]. Paclitaxel (PTX) is a diterpene found in the bark and branches of taxane plants. It was the first natural phytochemical approved by the FDA and has been considered to be the most effective natural anticancer drug [19]. Studies have found that paclitaxel can promote microtubule assembly and maintain microtubule stability, leading to the inability of cells to form spindles and spindle fibres during mitosis, thereby inhibiting cell division, proliferation and the subsequent growth of cancer cells [20]. Liposomes have the advantages of good biocompatibility, a simple preparation process, and fewer toxic effects and side effects and are widely used in gene transfection, drug delivery and in vivo imaging [21]. Our previous findings and other related studies have shown that nanobubbles with a lipid shell can enter tumour tissues through tumour blood vessels and concentrate therein for a long period of time [22]. Moreover, ultrasound-targeted nanobubble destruction (UTND) provides an effective way to direct drugs to specific parts of the body, effectively increasing the drug content in the tumour area and reducing potential side effects, thereby contributing effectively to the treatment of tumours. The underlying mechanisms may be associated with ultrasound cavitation effects [23–27]. The cavitation effect can cause reversible pores to appear on the cell membrane, resulting in increased permeability of the cell membrane, and enhance the entry of anticancer drugs into tumour cells. Based on these observations, along with the advantage of the strong affinity of the lipid shell for ICG and paclitaxel, multifunctional liposome nanobubbles (ICG-PTX NBs) containing both ICG and the chemotherapeutic drug paclitaxel were constructed to study their application in the ultrasound (US), fluorescence (FL) and photoacoustic (PA) imaging of prostate cancer xenografts and simultaneously analyse their effects on the inhibition of prostate cancer growth under ultrasound irradiation. This would offer avenues for the development of safe and stable nanobubbles with multimodal imaging functions as well as integrated diagnosis and treatment for prostate cancer.

## Materials and methods

### Reagents

Foetal bovine serum (FBS), Dulbecco’s modified Eagle’s medium (DMEM), and trypsin were purchased from BI, the Netherlands. 1,2-Dipalmitoyl-glycero-3-phosphate (DPPA), 1,2-dipalmitoyl-sn-glycero-3-phosphoglycerol (DPPG), 1,2-dipalmitoyl-sn-glycero-3-phosphatidylethanolamine (DPPE) and 1,2-dipalmitoyl-sn-glycero-3-phosphocholine (DPPC) were purchased from Corden Pharma, Switzerland. 1,2-Distearoyl-sn-glycero-3-phosphoethanolamine-PEG-2000 (DSPE-PEG2000) was purchased from NANOCS, USA. CCK-8 reagents and the TUNEL kit were purchased from Shanghai Biyuntian Biotechnology Co., Ltd. Paclitaxel was procured from Beijing Suolaibao Co., Ltd. Indocyanine Green was purchased from Sigma, USA. Perfluoropropane was purchased from Tianjin Institute of Physics and Chemistry. The aspartate aminotransferase test kit, phenylalanine aminotransferase test kit, serum creatinine test kit and blood urea nitrogen test kit were procured from Jiancheng Bioengineering Institute, Nanjing, China. Male nude mice were purchased from Beijing Huakangfu Biotechnology Co., Ltd.

### Cell culture and establishment of xenograft models

The human prostate cancer cell line PC-3 was kindly provided by the Stem Cell Bank, Chinese Academy of Sciences. Cells were cultured in DMEM containing 10% FBS, 100 U/mL penicillin and 100 μg/mL streptomycin at 37 °C with 5% CO_2_. Cells in the exponential growth phase were digested with 0.25% trypsin and passaged or used for experiments. A 200 µL suspension of 5 × 10^6^/mL PC-3 cells was injected into the dorsal side of 4 to 5-week-old male nude mice. Animal experiments were performed when the tumour volume reached 100 mm^3^ (tumour volume = a × b^2^/2 where a is the long diameter and b is the short diameter). All animal experimental protocols were approved by the Animal Ethics Committee of the Third Military Medical University.

### Preparation and characterization of ICG-PTX NBs

According to the ratio 3:3:3:1:1, a total of 11 mg of DPPC, DPPE, DPPG, DPPA, and DSPE-PEG2000 as well as 2 mg of paclitaxel and 500 µg of ICG were dissolved in 1000 µL of PBS/glycerol solution and heated for 20 min at 45 °C. The contents were transferred to another vial, the air in the vial was replaced using perfluoropropane, and the vial was then shaken in an ST amalgam capsule blender (AT&M, China) for 90 s and allowed to stand overnight at 4 °C. All the liquid in the vial was then transferred to a 10 mL centrifuge tube to be centrifuged at 300 rpm for 3 min, and the intermediate emulsion was collected into an Eppendorf tube to finish the preparation of ICG-PTX NBs. Another 11 mg of lipids was used to prepare blank nanobubbles following the above steps (Blank NBs).

The concentrations of ICG-PTX NBs and Blank NBs were calculated using a haemocytometer. The particle size, particle size distribution, and surface zeta potential were measured with a Zetasizer nano ZS90 particle size detector (Malvern, UK). Each measurement was repeated three times. The shape, size and distribution of the nanobubbles were observed by optical microscopy (Olympus, Japan) and transmission electron microscopy (JEOL, Japan). The nanobubbles were stored at 4 °C, and changes in the particle size and fluorescence intensity of ICG-PTX NBs were measured on days 0, 1, 3, 5, and 7 to analyse the stability of the ICG-PTX NBs. An ultraviolet–visible spectrophotometer (Thermo Fisher, USA) was used to obtain the UV absorption spectra of ICG-PTX NBs, Blank NBs, ICG and double distilled water. The standard curves of ICG and PTX were plotted, and the encapsulation efficiency (EE) and drug loading efficiency (LE) of ICG and PTX in ICG-PTX NBs were calculated. Calculation of EE and LE:

$${\text{EE}}\, = \,\left( {{\text{amount of ICG encapsulated in nanobubbles}}/{\text{total amount of ICG added}}} \right)\, \times \, 100\%$$

$${\text{LE }} = \left( {{\text{amount of ICG encapsulated in nanobubbles/the total amount of lipids used for the preparation of the nanobubbles}}} \right) \times 100\%.$$

### Haemolytic action of ICG-PTX NBs

After the microhematocrit blood tube was inserted into the inner corner of the eye, fresh blood was collected in an anticoagulant tube, and transferred to a 10 mL centrifuge tube. A 3 × volume of PBS was added, and the blood samples were centrifuged at 2000 rpm for 10 min to remove the supernatant. This washing was repeated 3 times until the supernatant was colourless and transparent. An appropriate amount of red blood cells (RBCs) and PBS were used to prepare a 2% RBC suspension followed by incubation with 1.0 × 10^8^/mL ICG-PTX NBs and Blank NBs for 1 h. Double distilled water was used as a positive control, and PBS was used as a negative control. The absorbance at 545 nm was measured by an ultraviolet–visible spectrophotometer, and the haemolysis rate was calculated using the following formula:

[(sample absorbance value − absorbance value of the negative control group) / (absorbance value of the positive control group − absorbance value of the negative control group)] × 100%.

### In vitro ultrasound, photoacoustic and fluorescence imaging using ICG-PTX NBs

Different concentrations of ICG-PTX NBs were placed in a cavity model made of 1% agarose gel (1.0 × 10^8^/mL, 5.0 × 10^7^/mL, 1.0 × 10^7^/mL, 5.0 × 10^6^/mL, and 1.0 × 10^6^/mL), and the corresponding ultrasound images in B-mode were acquired using a Vevo 2100 small animal ultrasound imaging system (VisualSonics, Canada) (centre frequency 18 MHz, gain 40 dB). Then, 1.0 × 10^8^/mL ICG-PTX NBs were mechanically blasted using ultrasonic waves of high mechanical index, and the ultrasonic images before and after blasting were analysed. The DFY-type diagnostic instrument for the quantitative analysis of ultrasonic images (Chongqing Institute of Ultrasound Molecular Imaging, China) was used to quantitatively assess the images and calculate the grey value for statistical analysis. The imaging parameters of the Vevo LAZR photoacoustic imager (VisualSonics, Canada) were set (centre frequency 21 MHz, gain 40 dB), and the optimal photoacoustic excitation wavelength for ICG-PTX NBs was measured by full wavelength scanning. Different concentrations of ICG-PTX NBs (1.0 × 10^8^/mL, 5.0 × 10^7^/mL, 1.0 × 10^7^/mL, 5.0 × 10^6^/mL, and 1.0 × 10^6^/mL) were placed in a cavity model made from 1% agarose gel for photoacoustic imaging at a wavelength of 825 nm, and a Vevo LAZR photoacoustic imager was used to collect photoacoustic images and quantitatively analyse the photoacoustic signals. ICG-PTX NBs (1.0 × 10^8^/mL) and ICG solution (0.35 mg/mL) with equal concentrations of ICG and double distilled water were added to Eppendorf tubes and then placed in the IVIS Spectrum living animal imaging system (PerkinElmer, USA). Under irradiation conditions at an excitation wavelength of 740 nm and emission wavelength of 820 nm, the Eppendorf tubes were scanned, and their fluorescence intensities were quantified.

### In vivo ultrasound, photoacoustic and fluorescence imaging using ICG-PTX NBs

When the volume of the subcutaneous tumour reached approximately 1 cm^3^, tumour blood flow was observed using ultrasound examination, and tumours with rich blood flow were selected. After anaesthetizing with isoflurane, the nude mice were fixed in the prone position and scanned using a Vevo 2100 small animal ultrasound imaging system and an MS250 high frequency probe. The sections with the optimal imaging effect were selected, and the optimal values of imaging parameters were determined (centre frequency of the probe 18 MHz, gain 40 dB). After injecting 200 µL of ICG-PTX NBs (1.0 × 10^8^/mL) through the orbital vein, the ultrasound imager was used to continuously acquire images at different time points after the injection of the contrast agent, and dedicated software (Vevo 2100 onboard software, VisualSonics, Canada) was used to analyse the time-intensity curve. After the contrast-enhanced echo had subsided, the “burst” button was used to blast the residual contrast agent. Blank NBs (200 µL, 1.0 × 10^8^/mL) were injected in the same way after the echo had completely subsided.

Chloral hydrate (4%, 0.20 mL/20 g) was intraperitoneally injected into nude mice as anaesthesia. The nude mice were fixed in the prone position, and the values of imaging parameters were adjusted (laser wavelength 825 nm, central frequency of the probe 21 MHz, gain 40 dB). The Vevo LAZR photoacoustic imager was used to collect photoacoustic images of the tumour before injection of the contrast agent. A total of 200 µL of ICG-PTX NBs (1.0 × 10^8^/mL) and ICG solution (0.35 mg/mL) of equal concentration were injected into the orbital vein. The photoacoustic imager was used to observe and store the dynamic images. Quantitative analysis of the photoacoustic signal in the region of interest was performed, and the time-photoacoustic signal intensity curve was plotted.

After anaesthetizing with isoflurane, the nude mice were fixed in the lateral position, and the parameters of the IVIS Spectrum living animal imaging system (with excitation and emission wavelengths set to 740 nm and 820 nm, respectively) were adjusted to collect fluorescence images of the nude mice before injection of the contrast agents. A total of 200 µL of ICG-PTX NBs (1.0 × 10^8^/mL) and ICG solution (0.35 mg/mL) of equal concentration were injected into the nude mice through the orbital vein, and fluorescence images were collected at different time points (3 min, 5 min, 10 min, 15 min, 30 min, and 60 min) for quantitative analysis of the metabolism of ICG-PTX NBs and ICG in tumour-bearing mice. Two hours after injection of the contrast agents, the animals were sacrificed, and the heart, liver, spleen, lung, kidney and tumour tissues were isolated for fluorescence imaging. The fluorescence intensity of each organ was quantitatively analysed by IVIS fluorescence analysis software to determine the level of residual ICG-PTX NBs in different tissues and organs.

### Effect of indocyanine green on the cavitation of nanobubbles

After cells in the logarithmic growth phase were seeded in a 96-well plate and cultured for 24 h, they were divided into 8 groups, namely, the blank nanobubbles group (Blank NBs), indocyanine green nanobubbles group (ICG NBs), paclitaxel nanobubbles group (PTX NBs), indocyanine green and paclitaxel nanobubbles group (ICG-PTX NBs), blank nanobubbles plus ultrasound irradiation group (Blank NBs + US), indocyanine green nanobubbles plus ultrasound irradiation group (ICG NBs + US), paclitaxel nanobubbles plus ultrasound irradiation group (PTX NBs + US), indocyanine green and paclitaxel nanobubbles plus ultrasound irradiation group (ICG-PTX NBs + US). The preparation method of the ICG NBs and PTX NBs was the same as that of the ICG-PTX NBs. Appropriate amounts of Blank NBs, ICG NBs, PTX NBs, and ICG-PTX NBs at concentrations of 1.0 × 10^8^/mL, 5.0 × 10^7^/mL, 1.0 × 10^7^/mL, and 5.0 × 10^6^/mL were added to the cells. After ultrasound irradiation intervention (1 W/cm^2^ irradiation for 20 s), cells were cultured for another 24 h, the optical density value (OD value) of each group was determined by CCK-8 assay, and the inhibition rate of cell proliferation of each group was calculated according to the following the formula:

$$\begin{aligned} {\text{inhibition rate of cell proliferation }}\left( \% \right)\, & = \,[({\text{OD experimental group}} - {\text{OD blank group}}) \\ & /({\text{OD control group}} - {\text{OD blank group}})]\, \times \, 100\% . \\ \end{aligned}$$

After the cells in the logarithmic growth phase were inoculated in a 6-well plate and cultured for 24 h, treatments were given following the above groupings, and the cells were cultured for another 24 h. The cells were then collected to prepare a cell suspension with staining buffer and stained with fluorescein isothiocyanate (FITC)-labelled Annexin V (Annexin V-FITC) and propidium iodide (PI) for 5 to 15 min at room temperature in the dark. Flow cytometry (ACEA, USA) was used to analyse the apoptotic effects in each group and to further analyse the effects of ICG on the cavitation of the nanobubbles.

### Cytocompatibility assay of ICG-PTX NBs

PC-3 cells in the logarithmic growth phase were resuspended to prepare a single-cell suspension after digestion, inoculated into a 96-well plate at a density of 1 × 10^4^ cells/well, and cultured at 37 °C in an incubator with 5% CO_2_ and saturated humidity for 24 h. The cells were then divided into six groups: blank control group (PBS), ultrasound irradiation group (US), paclitaxel group (PTX), paclitaxel plus ultrasound irradiation group (PTX + US), ICG-PTX NBs group and ICG-PTX NBs plus ultrasound irradiation group (ICG-PTX NBs + US). Reagents were administered to the PTX group, PTX + US group, ICG-PTX NBs group, and ICG-PTX NBs + US group at the IC_50_ concentration. After drug administration, ultrasound irradiation at 1 W/cm^2^ for 20 s was administered to the US group, PTX + US group and ICG-PTX NBs + US group. After the stipulated intervention for each group, cells were cultured for another 24 h; then, the OD value of each group was determined by CCK-8 assay, and the rate of inhibition of cell proliferation was calculated. PC-3 cells in the logarithmic growth phase were inoculated into 6-well plates at 5 × 10^4^ cells/well and cultured for 24 h. After the intervention for each group, the cells were cultured for another 24 h. After digestion and centrifugation, 50,000–100,000 cells were collected and resuspended in Annexin V-FITC solution, followed by staining with Annexin V-FITC and PI for 5 to 15 min at room temperature in the dark. The apoptotic effects of each group were analysed by flow cytometry.

### Inhibition effects of ICG-PTX NBs on prostate tumour growth and their in vivo safety

When tumour volumes reached 100 mm^3^, the tumour-bearing nude mice were randomly divided into six groups (n = 5): PBS, US, PTX, PTX + US, ICG-PTX NBs, and ICG-PTX NBs + US groups. All treatment groups were administered drugs through orbital vein injection every 3 days. Nude mice in the PTX, PTX + US, ICG-PTX NBs and ICG-PTX NBs + US groups received paclitaxel at a total dose of 20 mg/kg, and the US group, PTX + US group and ICG-PTX NBs + US group were subjected to ultrasound irradiation at 1 W/cm^2^ for 60 s. Drugs were administered for over a period of 18 days. The tumour volumes and body weights of the mice were measured before each administration, and the tumour growth curve and changes in mouse body weight were plotted until the last administration. One day before treatment and 1 day after the end of treatment, the maximum sections of the tumours in each group were scanned by contrast-enhanced ultrasound with a dosage of 200 µL 1 × 10^8^/mL Blank NBs, and the tumour growth before and after treatment in each experimental group was recorded. After congestion of the retroorbital venous plexus, the microhematocrit blood tube were inserted into the corner of the eye, the blood was collected using an anticoagulation tube, and blood biochemical indicators such as aspartate aminotransferase (AST), phenylalanine aminotransferase (ALT), serum creatinine (CRE) and blood urea nitrogen (BUN) were analysed to assess the toxic effects and side effects of the drugs on liver and kidney function in the tumour-bearing mice. At the end of the experiment, the mouse’s head was held with the left hand, the mouse’s tail was grasped with the right hand, the nude mouse was sacrificed by spinal dislocation, and the tumour tissues were routinely fixed, embedded, sectioned and subjected to haematoxylin–eosin (H&E) and TUNEL immunohistochemical staining to observe the morphological changes and apoptosis in each group. In addition, H&E staining was performed in the heart, liver, spleen, lung, kidney, etc. tissues obtained from the mice in each experimental group, and the biosafety of the drugs was analysed.

### Statistical analysis

One-way ANOVA and paired t-tests were performed using the Social Pack for Social Sciences 22.0, and the analysed data are expressed as the mean ± standard deviation. The LSD test was used for comparisons between groups. P < 0.05 indicates a significant difference in statistical analysis; * indicates P < 0.05 and ** indicates P < 0.01.

## Results

### Characterization of ICG-PTX NBs

**Fig. 1:**
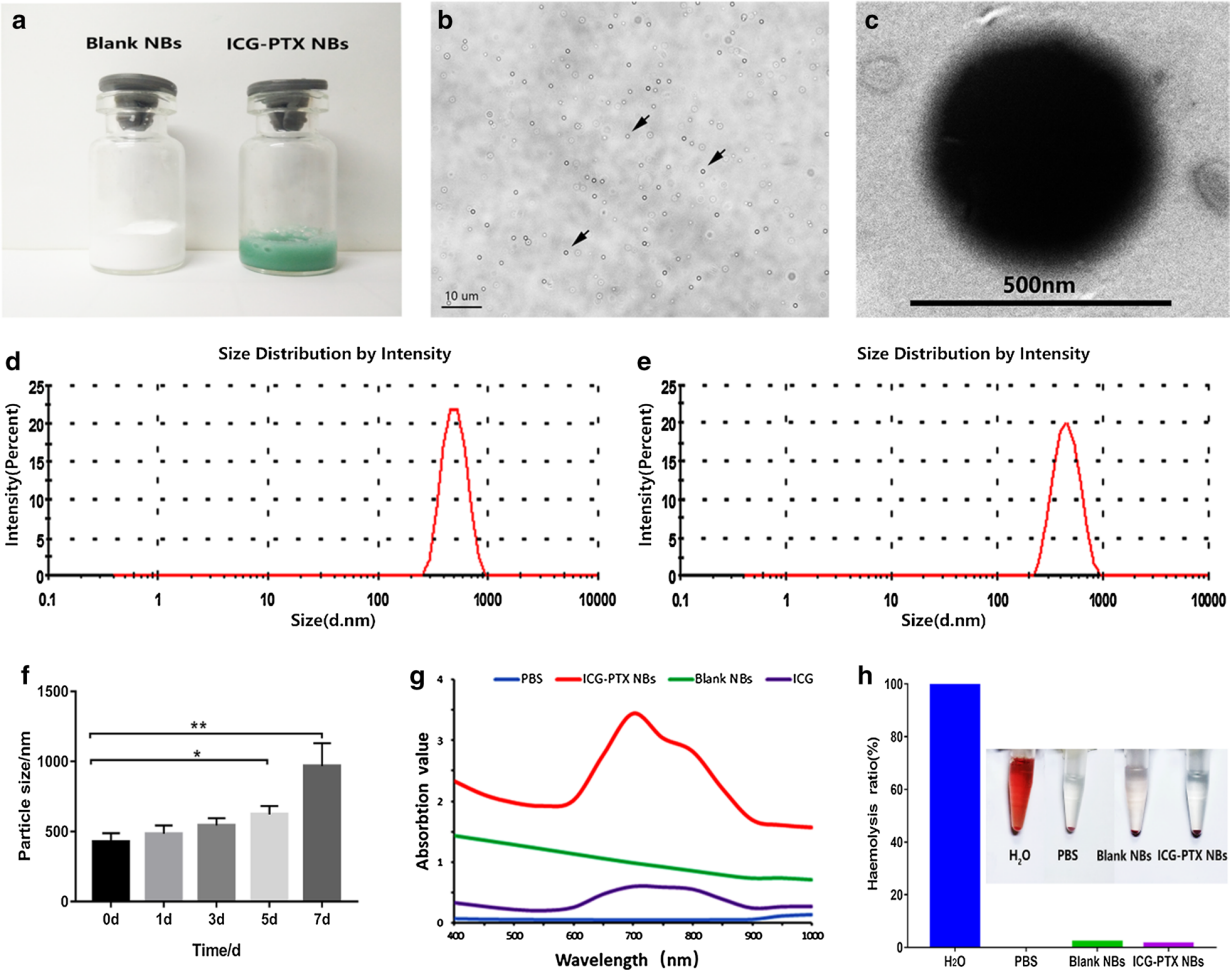
Basic characteristics of ICG-PTX NBs. **a** Photographs of Blank NBs and ICG-PTX NBs. **b** The distribution of ICG-PTX NBs under an optical microscope. **c** The morphology of ICG-PTX NBs under a transmission electron microscope. **d** The particle size of the Blank NBs. **e** The particle size of the ICG-PTX NBs. **f** Histogram of the particle size of ICG-PTX NBs changes over time. *P < 0.05, **P < 0.01. **g** UV–vis absorption spectra of Blank NBs, ICG-PTX NBs, ICG and H_2_O. (H) Photograph of haemolysis of erythrocytes incubated with Blank NBs and ICG-PTX NBs for 1 h and the haemolytic ratio measured by UV–vis spectrophotometry at 545 nm. Deionized water and PBS were used as positive and negative controls, respectively

The appearances of the ICG-PTX NBs and Blank NBs were observed (Fig. 1a), and the ICG-PTX NBs presented as a pale green emulsion, while the Blank NBs presented as a white emulsion, indicating that ICG was effectively encapsulated in ICG-PTX NBs. Under light microscopy, ICG-PTX NBs were found to be evenly distributed in size and shape without aggregation (Fig. 1b). The concentration of the ICG-PTX NBs was approximately (12.04 ± 2.02) × 10^8^/mL, the concentration of the Blank NBs was approximately (14.05 ± 2.79) × 10^8^/mL, and there was no significant difference between them (P > 0.05). Under transmission electron microscopy, the ICG-PTX NBs had a regular spherical shape with a clear and smooth surface (Fig. 1c). Furthermore, data from the Zetasizer nano ZS90 particle size detector suggested that the ICG-PTX NB particle size was 469.5 ± 32.87 nm with a polydispersity coefficient of 0.1733 ± 0.02 (Fig. 1e) whereas the Blank NBs had a particle size of 427.9 ± 35.39 nm and a polydispersity factor of 0.1193 ± 0.04 (Fig. 1d), and there was no significant difference between them (P > 0.05). The potentials of the ICG-PTX NBs and Blank NBs were − 21.70 ± 1.22 mV and − 23.63 ± 0.80 mV, respectively (Table 1). The strong negative zeta potential of the two kinds of nanobubbles is an important factor for the nanobubbles to avoid aggregation and remain stable. The particle sizes of ICG-PTX NBs at different time points (1, 3, 5, and 7 days) were 484.7 ± 34.54 nm, 544.6 ± 29.75 nm, 623.3 ± 34.09 nm, and 968.2 ± 94.11 nm, respectively. On the 5th day of storage, the particle size of ICG-PTX NBs was significantly different from the particle size on the day of preparation, indicating that ICG-PTX NBs have good stability within 5 days of preparation (Fig. 1f). The UV absorption spectrum showed that ICG-PTX NBs had a major absorption peak at 780 nm, indicating that ICG was successfully loaded into the ICG-PTX NBs (Fig. 1g). In addition, by plotting the ICG and PTX standard curves, it was found that the encapsulation efficiency of ICG in ICG-PTX NBs was 68% and the drug loading efficiency was 2.52%, while the encapsulation efficiency of PTX was 78.2%, and the corresponding drug loading efficiency was 11.59%.

**Table 1:** Physical characteristic parameters of the ICG-PTX NBs and Blank NBs

Nanobubbles	Concentration( × 10^8^/mL)	Particle size(nm)	Electric potential(mV)	PDI
ICG-PTX NBs	12.04 ± 2.02	469.5 ± 32.87	− 21.7 ± 1.22	0.1733 ± 0.02
Blank NBs	14.05 ± 2.79	427.9 ± 35.39	− 23.63 ± 0.80	0.1193 ± 0.04

There is no significant difference between the parameters (n = 3)

Haemolysis is an important criterion for evaluating the biocompatibility of nanobubbles in drug delivery. Haemolytic action is evaluated by determining the content of haemoglobin released during the contact of nanobubbles with red blood cells. Studies have shown that the haemolysis rate of biocompatible materials should be less than 5%, and the higher the haemolysis rate, the greater the damage to red blood cells [17]. Figure 1h shows that after red blood cells were incubated for 1 h with ICG-PTX NBs and Blank NBs, all the contents settled at the bottom of the tube, the supernatant was colourless and transparent, and the measured haemolysis rates were less than 5% for both samples. In the positive control group, the solution was red and clear, and there were no RBCs at the bottom of the tube. The experimental results showed that ICG-PTX NBs do not cause haemolysis.

### In vitro ultrasound, photoacoustic and fluorescence imaging using ICG-PTX NBs

**Fig. 2:**
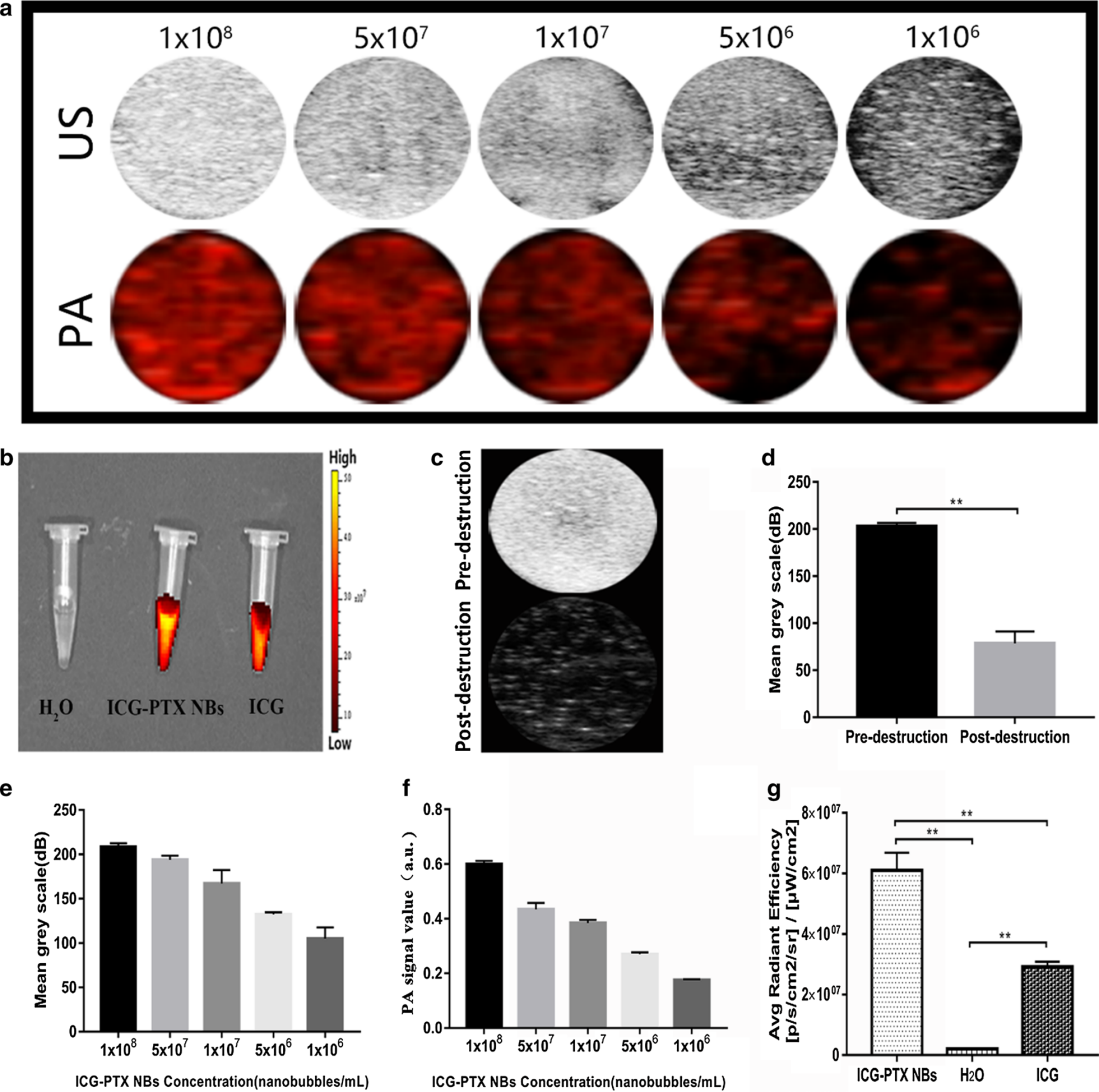
In vitro imaging of ICG-PTX NBs. **a** Ultrasound images and photoacoustic images of ICG-PTX NBs at different concentrations in vitro. **b** Fluorescence images of H_2_O, ICG-PTX NBs and ICG. **c** Ultrasound images of ICG-PTX NBs before and after destruction. **d** Imaging intensity quantification of ICG-PTX NBs before and after destruction, *P < 0.05, **P < 0.01. **e** Ultrasound imaging quantification of ICG-PTX NBs in vitro. **f** Photoacoustic imaging quantification of ICG-PTX NBs in vitro. **g** Fluorescence imaging quantification of H_2_O, ICG-PTX NBs and ICG in vitro, *P < 0.05, **P < 0.01

In the in vitro agarose gel model, the ultrasound imaging signal intensity of the ICG-PTX NBs was found to be positively correlated with their concentration (Fig. 2a–e). After ultrasound irradiation with a high mechanical index, the ultrasound imaging intensity of the ICG-PTX NBs was significantly reduced, indicating that ICG-PTX NBs can be destroyed by ultrasound with a high mechanical index (Fig. 2c–d). In vitro photoacoustic imaging showed that ICG-PTX NB could achieve photoacoustic imaging, and the photoacoustic image contrast was enhanced with increasing ICG-PTX NBs concentration (Fig. 2a–f). When the IVIS Spectrum living animal imaging system was used to scan the ICG-PTX NBs, ICG solution and double distilled water, the ICG-PTX NBs and ICG solution groups showed obvious fluorescence signals, and the fluorescence signal of the ICG-PTX NBs was markedly stronger than that of the ICG solution, whereas no significant fluorescence signal was seen in the double distilled water group (Fig. 2b–g). Together, the results of ultrasound, photoacoustic, and fluorescence imaging in the in vivo model indicate that ICG-PTX NBs have multimodal imaging capabilities.

### In vivo ultrasound, photoacoustic and fluorescence imaging using ICG-PTX NBs

Analysis and comparison of ultrasound molecular imaging effects of ICG-PTX NBs and Blank NBs in PC-3 xenografts.

**Fig. 3:**
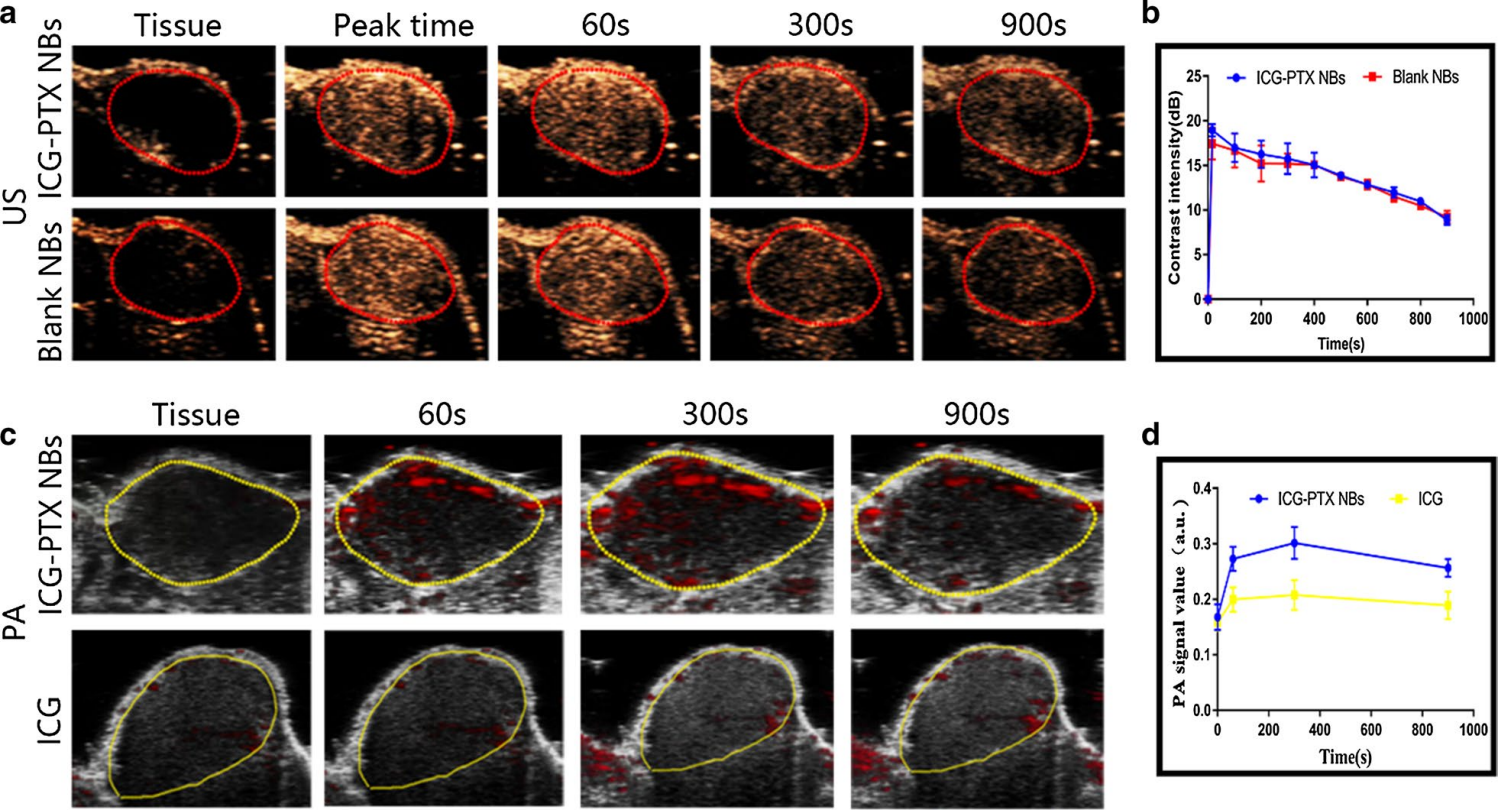
In vivo imaging characteristics of nude mice bearing PC-3 tumours after orbital vein injection of ICG-PTX NBs. **a** Ultrasound images of Blank NBs and ICG-PTX NBs in transplanted tumour tissues. **b** Time-ultrasound intensity curves of nanobubbles in transplanted tumour tissues (n = 3). **c** Photoacoustic images of ICG-PTX NBs and ICG in transplanted tumour tissues. **d** Time-photoacoustic intensity curves of ICG-PTX NBs and ICG in transplanted tumour tissues (n = 3)

The time taken to reach peak intensity for the ICG-PTX NBs was 18.10 ± 0.8227 s, the peak intensity was 17.45 ± 0.7765 dB, the duration was 22.70 ± 0.4821 s and the area under the curve was 13,124 ± 209.3000 dB s, which were not significantly different from those of the Blank NBs (P > 0.05) (Table 2). The time-intensity curve showed no significant difference in ultrasound molecular imaging effects between ICG-PTX NBs and Blank NBs in PC-3 xenografts (Fig. 3b).

**Table 2:** Ultrasound parameters of the ICG-PTX NBs and blank NBs in nude mice bearing PC-3 tumours

Contrast agents	Peak time/s	Peak intensity/dB	Duration time/min	AUC/dB.s
Blank NBs	18.11 ± 0.4944	17.00 ± 0.2233	22.56 ± 0.4812	13228 ± 130.3000
ICG-PTX NBs	18.10 ± 0.8227	17.45 ± 0.7765	22.70 ± 0.4821	13124 ± 209.3000

There was no significant difference between the parameters (n = 3)

Analysis and comparison of the photoacoustic imaging effects of the ICG-PTX NBs and ICG solution in PC-3 xenografts.

After injection of ICG-PTX NBs, the photoacoustic (PA) signal in the xenograft area gradually increased, and the photoacoustic imaging effect was observed to be the best 5 min after injection. The PA signal in the tumour area was still enhanced 15 min after injection. There was no significant increase in photoacoustic signals at each time point in the xenograft area of the nude mice injected with ICG solution (Fig. 3c). The time-intensity curve indicates that ICG-PTX NBs have considerable photoacoustic imaging capabilities (Fig. 3d).

In vivo fluorescence imaging effects of the ICG-PTX NBs and ICG solution in PC-3 xenografts.

**Fig. 4:**
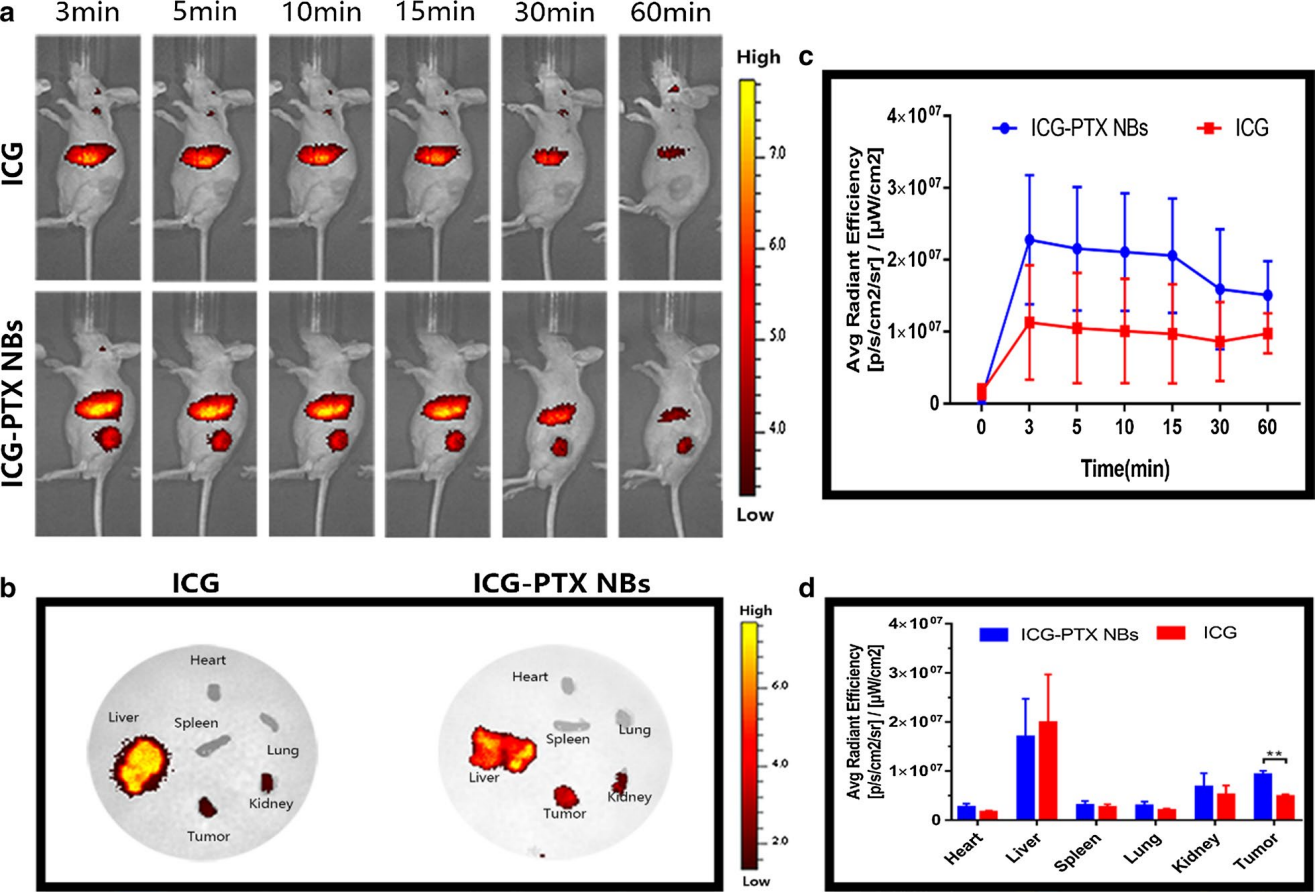
In vivo fluorescence imaging characteristics of nude mice bearing PC-3 tumours after orbital vein injection of ICG and ICG-PTX NBs. **a** Near-infrared fluorescence images of tumour-bearing nude mice. **b** NIR fluorescence images of major organs and tumours 2 h post-injection of ICG and ICG-PTX NBs. **c** Time-fluorescence intensity curve in the tumour region at different time points, the data are shown as mean ± SD (n = 3). **d** Semiquantitative biodistribution of ICG and ICG-PTX NBs in nude mice determined by the averaged fluorescence intensity of each organ. The data are shown as the mean ± SD (n = 3), *P < 0.05, **P < 0.01

Three minutes after ICG-PTX NBs injection, obvious fluorescence signals were observed in the liver and tumour site. After 5 min, the fluorescence signal in the tumour site began to decay gradually, and after 60 min, only a small amount of fluorescence signal remained. Within 60 min of ICG solution injection, obvious fluorescence signals were observed in the liver, but no obvious fluorescence signal was observed in the tumour site (Fig. 4a). The time-fluorescence intensity curve showed a significant difference in the fluorescence imaging effect between the ICG-PTX NBs and ICG solution in PC-3 xenografts (P < 0.05) (Fig. 4c). In addition, the vital organs (heart, liver, spleen, lung, kidney) and tumour tissues of tumour-bearing nude mice were collected for ex vivo fluorescence imaging 2 h after injection. The results showed that the vital organs and tumour tissues of both the ICG-PTX NBs group and the ICG solution group had different fluorescence signals intensities, with the liver and kidney being the most marked. In addition, the fluorescence signal of tumour tissues in the ICG-PTX NBs group was significantly stronger than that in the ICG solution group (P < 0.05) (Fig. 4b–d).

### Effects of ICG on nanobubble cavitation

**Fig. 5:**
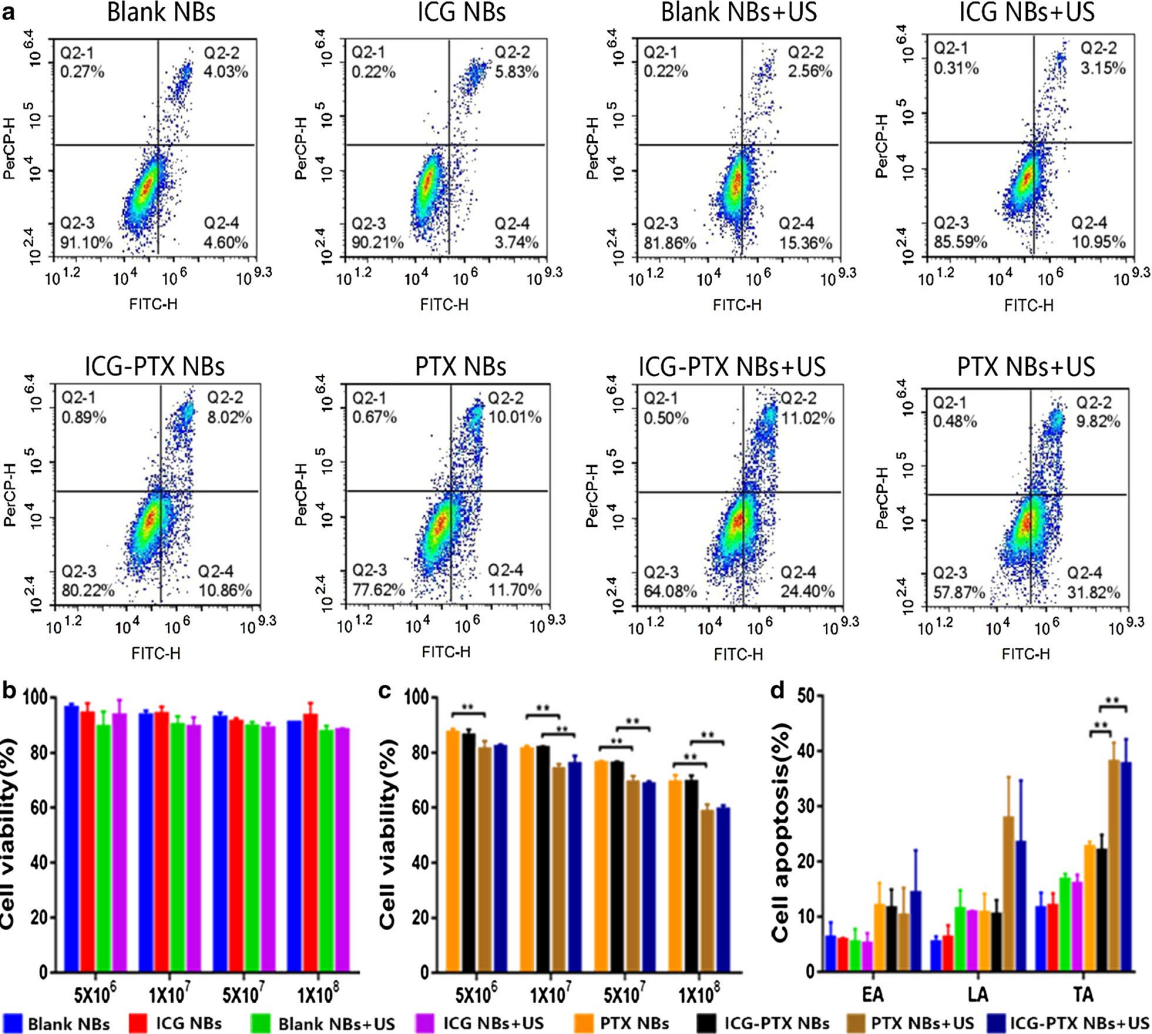
The effects of ICG on nanobubble cavitation in vitro. **a** FCM images in each group. **b** Cell viability of PC-3 cells treated with Blank NBs or ICG NBs with or without US irradiation. **c** Cell viability of PC-3 cells treated with PTX NBs or ICG-PTX NBs with or without US irradiation. **d** Apoptosis of PC-3 cells treated with Blank NBs, ICG NBs, PTX NBs and ICG-PTX NBs with or without US irradiation. EA: early apoptosis, LA: late apoptosis, TA: total apoptosis. The data are shown as the mean ± SD (n = 3), *P < 0.05, **P < 0.01

A CCK-8 assay was utilized to assess the status of PC-3 cell proliferation after treatment with four different concentrations of Blank NBs, ICG NBs, PTX NBs and ICG-PTX NBs with or without ultrasound irradiation. Before ultrasound irradiation, there was no significant difference in cell viability between the different concentrations of the Blank NBs group and ICG NBs group (P > 0.05) or the PTX NBs group and ICG-PTX NBs group (P > 0.05). After ultrasound irradiation, no significant difference in cell viability was observed between the Blank NBs + US group and the ICG NBs + US group (P > 0.05) or between the PTX NBs + US group and the ICG-PTX NBs + US group. There were significant differences in cell viability before and after ultrasound irradiation in the PTX NBs and ICG-PTX NBs groups (P < 0.05) (Fig. 5b, c). The results of the CCK-8 assay showed that ICG had no significant effect on the cytotoxicity of the nanobubbles before or after ultrasound irradiation. Flow cytometry was used to analyse the cell apoptosis status of PC-3 cells after treatment with Blank NBs, ICG NBs, PTX NBs and ICG-PTX NBs with or without ultrasound irradiation. The flow cytometry results were consistent with those of the CCK-8 assay (Fig. 5a–d). These observations indicate that loading ICG has no significant effect on the therapeutic functions of nanobubbles, and under the action of ultrasonic irradiation, ICG does not have a differential effect on the cavitation of the nanobubbles.

### Cytocompatibility assay of the ICG-PTX NBs

**Fig. 6:**
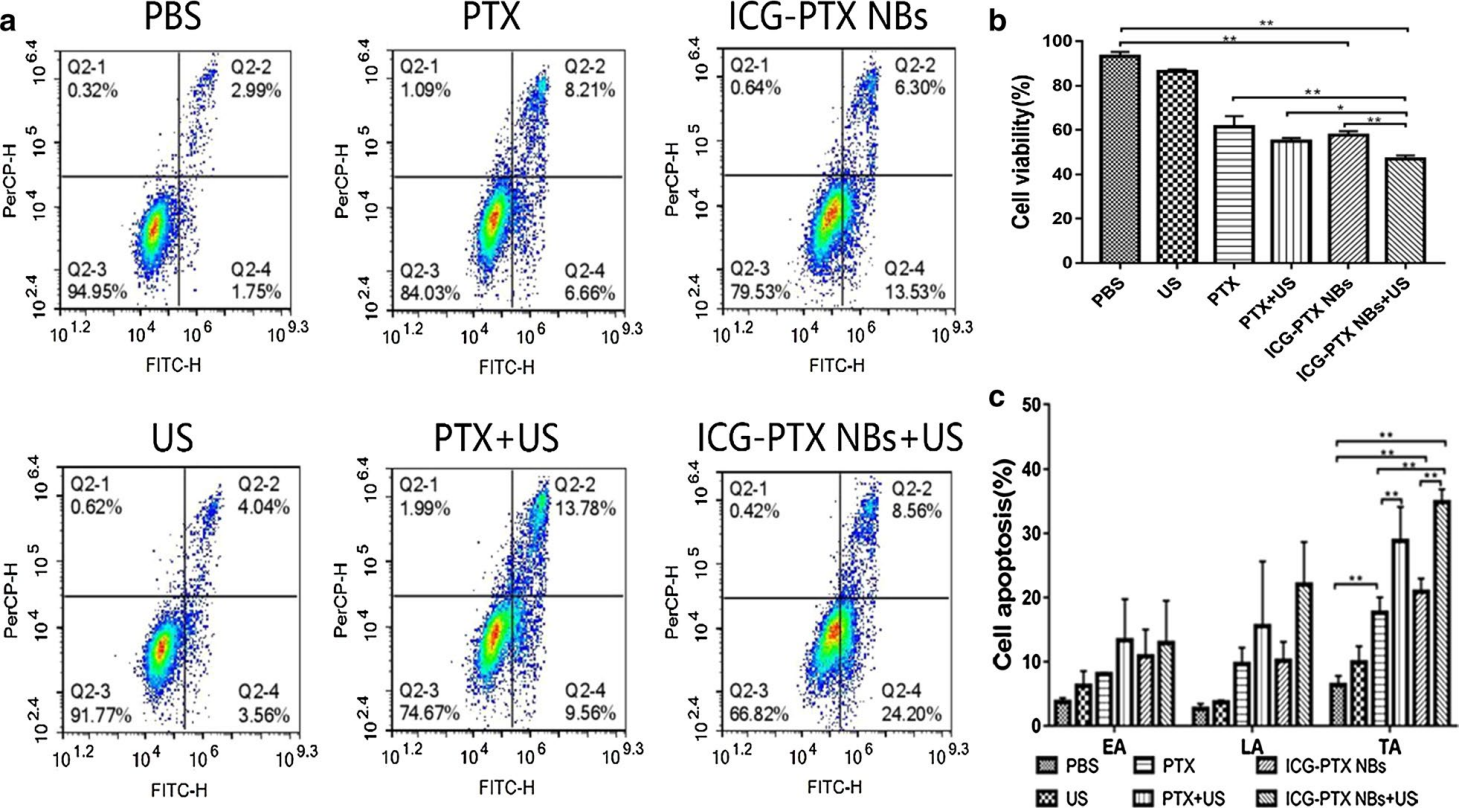
In vitro cytotoxicity and apoptosis. **a** FCM images of PC-3 cells treated with PBS, US, PTX, PTX + US, ICG-PTX NBs and ICG-PTX NBs + US. **b** Cell viability of PC-3 cells treated with PBS, PTX, or ICG-PTX NBs with or without US irradiation. **c** Apoptosis of PC-3 cells treated with PBS, PTX, or ICG-PTX NBs with or without US irradiation. EA: early apoptosis, LA: late apoptosis, TA: total apoptosis. Each bar represents the mean ± SD of three experiments. *P < 0.05, **P < 0.01

A CCK-8 assay was used to evaluate the inhibitory effect on PC-3 cell proliferation in the different treatment groups. The results showed that the US group, PTX group, PTX + US group, ICG-PTX NBs group, and ICG-PTX NBs + US group had different degrees of inhibitory effects on cell proliferation. PC-3 cells in the ICG-PTX NBs + US group had the lowest survival rate, which was significantly different from that of the ICG-PTX NBs group (P < 0.05). There was no significant difference in the cell survival rate between the PTX group and the ICG-PTX NBs group (P > 0.05), but the cell survival rate of the ICG-PTX NBs + US group was significantly lower than that of the PTX + US group (P < 0.05) (Fig. 6b). An Annexin V-FITC/PI double staining kit was used to evaluate the apoptosis of PC-3 cells after different treatments. The total apoptotic effects of the experimental groups were ranked as follows: ICG-PTX NBs + US > PTX + US > ICG-PTX NBs > PTX > US > PBS. These results are consistent with those of the CCK-8 assay (Fig. 6a–c). The total apoptosis rate of the ICG-PTX NBs + US group was the highest, which was significantly different from that of the ICG-PTX NBs group (P < 0.05), indicating that ultrasound irradiation can promote apoptosis. There was no obvious difference between the ICG-PTX NBs group and PTX group, and the apoptosis rates of the ICG-PTX NBs + US group and PTX + US group were found to be significantly higher than that of the US group, indicating that ICG-PTX NBs stably carry PTX and have an antitumour effect.

### Inhibitory effects of ICG-PTX NBs on the growth of PC-3 xenografts in nude mice

**Fig. 7:**
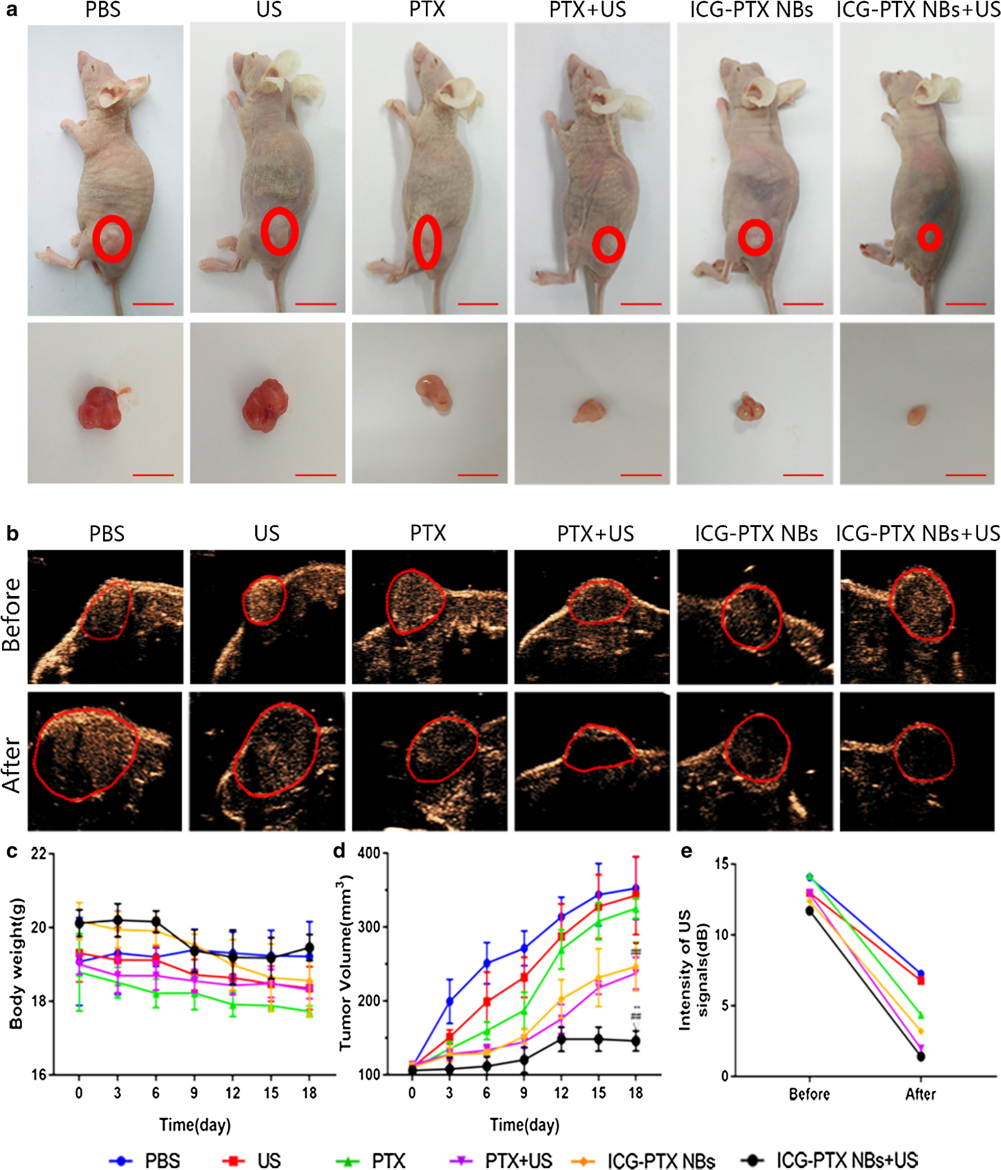
In vivo antitumour efficacy in a PC-3 xenograft mouse model. **a** Tumour volumes in the various treatment groups. The red circles represent the xenograft areas. Scale, 1 cm. **b** Contrast-enhanced imaging of the largest section of the transplanted tumour before and after treatment in the various treatment groups, in which the red circle indicates the area of the transplanted tumour. **c** Changes in body weight in the various treatment groups. **d** Tumour growth curve in the various treatment groups. **e** Changes in the ultrasonic signal intensity of the maximum sections of xenografts in the six groups. The data are shown as the mean ± SD (n = 3), compared to PBS, *P < 0.05, **P < 0.01; compared to PTX, ^#^P < 0.05, ^##^P < 0.01; compared to ICG-PTX NBs, ^∇^P < 0.05, ^∇∇^P < 0.01

**Fig. 8:**
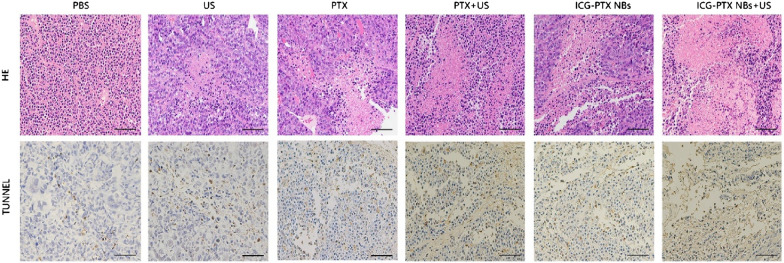
H&E- and TUNEL-stained images of tumour tissue sections from xenograft-bearing mice receiving different treatments after 18 days of treatment. H&E assay results revealed blue staining of the cell nuclei and red staining of the cytoplasmic and extracellular matrix. TUNEL assay analysis showed that the brown staining of the cell nuclei indicated apoptosis- and proliferation-positive tumour cells, whereas blue staining of the cell nuclei indicated apoptosis- and proliferation-negative tumour cells. Scale, 100 μm

**Fig. 9:**
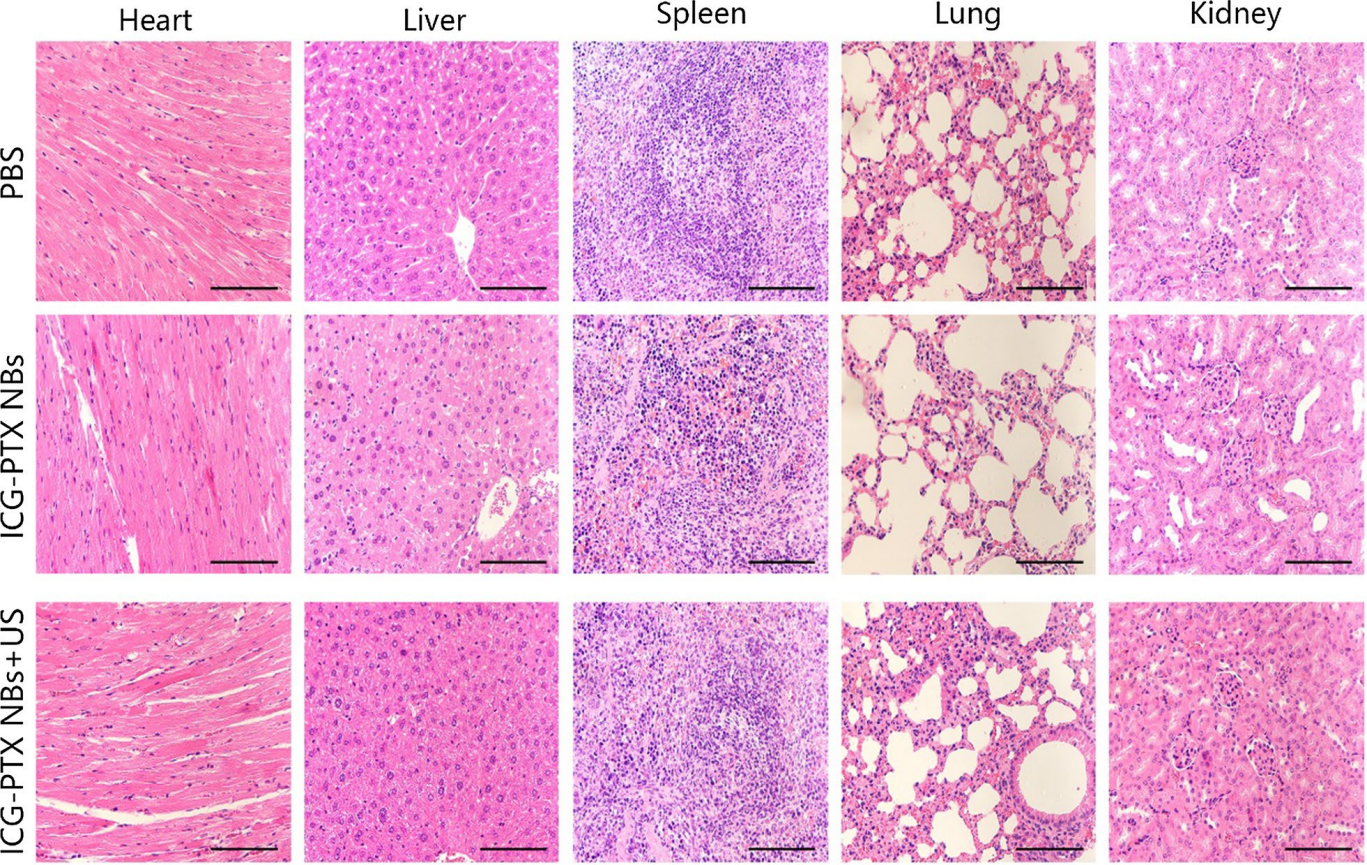
H&E stained images of sliced major organs, including the heart, liver, spleen, lung, and kidney, collected from mice sacrificed after injection of ICG-PTX NBs with or without US irradiation. Scale, 100 μm

**Fig. 10:**
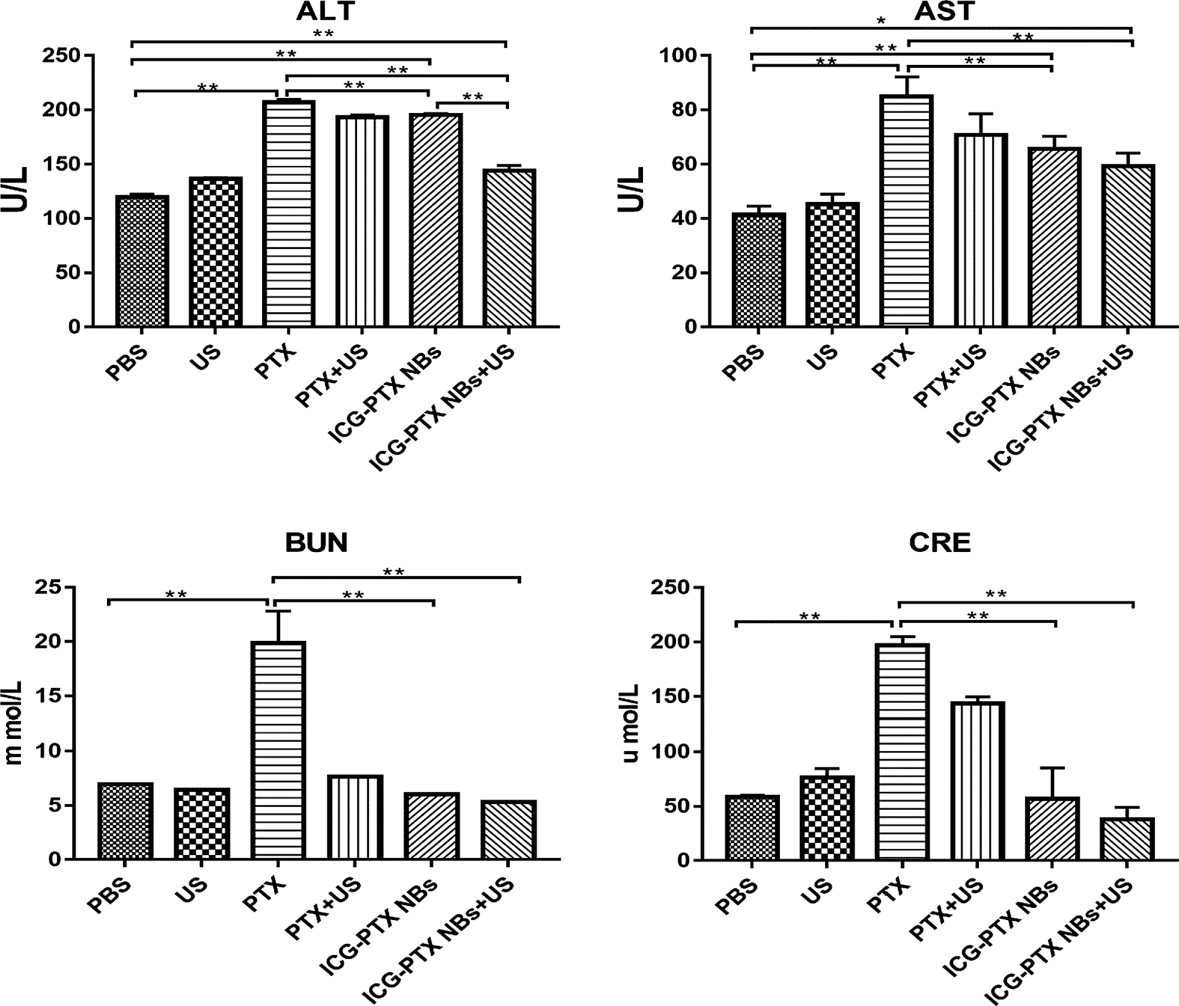
Blood biochemistry measurements of tumour-bearing mice after injection of ICG-PTX NBs with or without US irradiation. Each bar represents the mean ± SD of three experiments. *P < 0.05, **P < 0.01

The tumour volume changes in tumour-bearing nude mice (six groups) were observed after an 18-day treatment period with PBS, US, PTX, PTX + US, ICG-PTX NBs and ICG-PTX NBs + US. Among these, the ICG-PTX NBs + US group showed no significant tumour growth, while the remaining five groups had tumours with varying degrees of growth. The tumour growth curve showed that the volume of tumours in the control group increased 3.5-fold after the end of treatment; the ICG-PTX NBs group increased 2.5-fold, and the ICG-PTX NBs + US group grew the slowest (1.5-fold). The tumour volume was significantly smaller in the ICG-PTX NBs group and the ICG-PTX NBs + US group than in the PBS and PTX groups (P < 0.05), and there was also a significant difference in tumour volume between the ICG-PTX NBs group and the ICG-PTX NBs + US group (P < 0.05) (Fig. 7d). Previous studies have shown that contrast-enhanced ultrasound images can reflect the internal necrosis of xenograft tumours [4]. Figure 7b shows that there were unfilled anechoic areas in the xenograft tumour centre in the PTX group, PTX + US group, ICG-PTX NBs group, and ICG-PTX NBs + US group, indicating different degrees of necrosis inside the xenograft tumours in each treatment group, of which the ICG-PTX NBs + US group had the most severe necrosis. Our observations indicate that tumour necrosis was promoted by ultrasound irradiation, producing a significant therapeutic effect. Furthermore, TUNEL staining was used to assess the number of apoptotic cells in each treatment group. The number of apoptotic cells in the ICG-PTX NBs + US group was the highest. H&E staining showed cell necrosis in each treatment group, which was found to be most significant in the ICG-PTX NBs + US group (Fig. 8). In addition, H&E staining of vital organs, such as the heart, liver, spleen, lung and kidney, showed that the organs from each treatment group had normal cellular morphology, clear structure and no apparent histological changes (Fig. 9). The results of blood biochemical analysis showed that the PTX group had the highest levels of ALT, AST, BUN and CRE compared with the PBS control group, followed by the PTX + US group, the ICG-PTX NBs group and the ICG-PTX NBs + US group. The ALT, AST, BUN and CRE values of the PTX group were significantly different from those of the PBS group (P < 0.05). Only the ALT and AST values of the ICG-PTX NBs group and the ICG-PTX NBs + US group were significantly different from those of the PBS group (P < 0.05), and the ALT, AST, BUN and CRE values of the ICG-PTX NBs group and the ICG-PTX NBs + US group were lower than those of the PTX group and were statistically significant (P < 0.05). These data show that liver function (ALT, AST) in the ICG-PTX NBs group and ICG-PTX NBs + US group were different from that in the normal control group (P < 0.05). Moreover, the liver function and kidney function of the ICG-PTX NBs group and ICG-PTX NBs + US group were also significantly different from those of the PTX group (P < 0.05) (Fig. 10), indicating that ICG-PTX NBs and ICG-PTX NBs + US are less toxic than free PTX.

## Discussion

In recent years, prostate cancer has become one of the tumours with the highest growth rate of incidence. It is characterized by insidious onset, frequent bone metastasis and hormone dependence. Finding a method for the early diagnosis and effective treatment of prostate cancer has always been a focus and challenge in cancer research. Related treatment studies have shown that although endocrine therapy is effective in the initial treatment of prostate cancer by effectively controlling disease development in patients, the patient always enters the hormone-independent stage as time progresses. At this stage, treatment is based on chemotherapy. Paclitaxel chemotherapy combined with prednisone can not only effectively improve the clinical symptoms of prostate cancer but also relieve pain, thereby prolonging the survival time of patients. For these reasons, it has become the first choice for the treatment of hormone-independent prostate cancer [28]. However, akin to chemotherapy for other cancers, prostate cancer chemotherapy also poses problems such as toxicity and side effects on the liver, heart and kidney, being unamenable to monitoring in real-time if the drug reaches the target organ, and difficulty in locally concentrating the chemotherapeutic agents. Resolving these issues is the key link to improve prostate cancer chemotherapy [29].

Liposomes have the advantages of good biocompatibility and biodegradability and efficient loading of lipophilic and hydrophilic drugs. Therapeutic drugs can be loaded inside the double-layer lipid shells or lipid cores, or they can be attached to the surface of the liposomes, showing great advantages in drug delivery [30]. In our previous study, a lipid-shell nanobubble carrying androgen receptor double-stranded RNA (AR dsRNA) was constructed for androgen-independent prostate cancer, and was confirmed to accumulate in prostate cancer lesions and release AR dsRNA under ultrasound irradiation, thereby inhibiting the growth of prostate cancer. This shows that nanobubbles can be used as drug or gene carriers with strong penetrability and high drug loading efficiency [31]. However, since ultrasound imaging generates greyscale images by acoustic reflection, contrast-enhanced ultrasonography alone cannot sensitively display and monitor the aggregation and disappearance of drug-loaded nanobubbles in local lesions. Photoacoustic imaging is a new type of non-invasive and non-radiative imaging method that has been rapidly developed in recent years. It combines the high spatial resolution of ultrasonography and the high contrast of optical imaging to achieve visual dynamic imaging of the target tissue in a damage-free, real-time, multi-level and multi-contrast manner, providing a novel approach for the early detection and therapeutic monitoring of tumours. Multimodal imaging technology, combining ultrasound and photoacoustic methods, can overcome the shortcomings of the single imaging method discussed above. The combination of multiple imaging technologies can complement each other and is expected to solve the problems encountered in tumour diagnosis and post-treatment evaluation. The near-infrared fluorescent dye ICG can simultaneously achieve photoacoustic and fluorescence imaging, but it has drawbacks such as rapid elimination in vivo and poor stability in aqueous solution when used alone. Related studies have shown that the encapsulation of ICG into liposome carriers can improve its stability, prolong its duration in circulation, and increase its fluorescence and photoacoustic signal intensity [32]. Based on the advantages of lipid-shell nanobubbles that can encapsulate therapeutic drugs and be used for ultrasound/photoacoustic/fluorescent multimodal imaging, as well as the important role of paclitaxel in prostate cancer chemotherapy, this study integrated the superiority of lipid nanobubbles, multimodal imaging and the chemotherapeutic drug paclitaxel. We encapsulated both ICG and paclitaxel into the lipid shell to construct nanobubbles that can be used for ultrasound/photoacoustic/fluorescent multimodal imaging and integrated diagnosis and treatment, thereby generating new avenues for the diagnosis and treatment of prostate cancer.

In vitro fluorescence imaging experiments showed that ICG-PTX NBs had slightly stronger fluorescence intensity than ICG at the same ICG concentration because ICG binds to the lipid membrane and is completely and stably integrated in ICG-PTX NBs, indicating that the presence of lipids in ICG-PTX NBs is effective in stabilizing and increasing the fluorescence intensity of ICG, which is consistent with the findings of Kraft, John C et al. [33]. The fluorescence measurements illustrated that the fluorescence intensity of ICG in ICG-PTX NBs was still significantly higher than that of free ICG after 7 days, indicating that ICG was loaded in liposomes and that its fluorescence stability was significantly improved. In addition, in vitro photoacoustic and ultrasound imaging confirmed that with the increase in ICG-PTX NB concentration, the signal intensity of photoacoustic and ultrasonic imaging also increased, indicating that ICG-PTX NBs are of good use for ultrasound, fluorescence and photoacoustic imaging. In vivo imaging of prostate cancer xenografts in nude mice demonstrated that ICG-PTX NBs have the advantages of stable metabolism and safety in vivo and can accumulate rapidly at tumour sites in a short period of time and achieve long-term tumour monitoring through multimodal imaging functions such as ultrasound imaging, fluorescence imaging and photoacoustic imaging, with high contrast, high sensitivity and real-time imaging. Related studies have confirmed that the size of the nanomedicine is a key factor in passive targeting and drug accumulation during drug delivery and has an important impact on the therapeutic effect of drugs. Therefore, generally, large nanobubbles accumulate only near the vasculature whereas small nanobubbles can rapidly diffuse throughout the tumour matrix and provide better penetration. Our previous research results confirmed that a nanobubble particle size of 500 nm can rely on the EPR effect to smoothly enter the tumour tissue through the tumour neovascular wall and accumulate in the tumour tissue for a long time. However, drug delivery systems that rely on tumour EPR effects have limitations such as low drug utilization [34]. Under the action of ultrasound irradiation, the nanobubbles can undergo unstable expansion and intense collapse, resulting in temporary and permanent pores in the neovascular basement membrane of the tumour, which facilitates the entry of more nanobubbles from the tumour vessels into the tumour tissue [15, 35]. Studies by Rajeet Chandan et al. have shown that ultrasound irradiation can completely disintegrate the nanobubble core and the spherical structure constructed by the bound liposomes into lipid fragments, resulting in complete loss of the liposome double-layer integrity, hence triggering drug release [26]. The ICG-PTX NBs designed and constructed in this study are also lipid-shell nanobubbles with a particle size of 469.5 ± 32.87 nm. In vivo experiments have confirmed that they can enter the tumour tissue through the tumour neovascular wall, enhance their infiltration and accumulation in prostate cancer and achieve multimodal imaging and effective treatment of prostate cancer. Moreover, under the action of ultrasound irradiation, ICG and PTX in the liposome of the nanobubble shell can be effectively released into the tumour tissue in a controlled manner to kill prostate cancer cells in a close range with high efficiency. Further results showed that under the action of ultrasound radiation, the tumour volume inhibition and necrosis of tumour tissue in the ICG-PTX NBs group were enhanced compared with the groups that were not subjected to ultrasound radiation. This is primarily because cavitation mediates the enhancement of cell permeability and triggers the rupture of nanobubbles to release paclitaxel, and the synergy of the two further improves the antitumour effects of ICG-PTX NBs.

Compared to free drugs, nanodrug delivery systems based on liposome encapsulation are effective in enhancing bioavailability and reducing the side effects of antitumour drugs [36]. The results of this study show that compared to direct treatment with PTX alone, ICG-PTX NBs not only have significant antitumour effects in vitro and in vivo but they also do not cause any significant damage to vital organs such as the liver and kidney. Additionally, the release of drugs at tumour sites caused by cavitation effects reduces the systemic toxicity of paclitaxel. H&E staining confirmed that ICG-PTX NBs and ICG-PTX NBs + US caused no significant toxic effects or side effects on vital organs, such as the heart, liver, spleen, lung and kidney. Blood biochemical analysis showed that ICG-PTX NBs and ICG-PTX NBs + US with liposome encapsulation had lower toxic effects than PTX alone, further confirming that the ICG-PTX NBs constructed in this study can not only effectively kill tumour cells under ultrasound irradiation and inhibit tumour growth but are also safe without causing significant damage to important organs of the human body.

## Conclusions

As a multimodal contrast agent and carrier for chemotherapeutic drugs, the ICG-PTX NBs constructed in this study, while effectively achieving ultrasound/photoacoustic/fluorescence multimodal imaging, can significantly enhance PTX release and apoptosis of prostate cancer cells under the synergistic effects of low intensity ultrasound irradiation, inhibit tumour growth, and display appreciable biosafety. The successful preparation and experimental study with ICG-PTX NBs provides new strategies and methods for multimodal imaging diagnosis and the close-range local chemotherapy of prostate cancer. However, ICG-PTX NBs are incapable of active targeting. Therefore, our future studies would involve strategies to construct ICG-PTX NBs that can target prostate cancer by actively and specifically binding to prostate cancer cells. This would allow targeted multimodal imaging of tumours and further enhance the killing effects in tumour cells under guided ultrasound irradiation.

## Data Availability

All data generated or analysed during this study are included in the article and additional file.

## Abbreviations

UTND: Ultrasound targeted nanobubble destruction
NBs: Nanobubbles
AST: Aspartate aminotransferase
ALT: Phenylalanine aminotransferase
CRE: Serum creatinine
BUN: Blood urea nitrogen
ICG: Indocyanine green
NIR: Near-infrared
PTX: Paclitaxel
US: Ultrasound
FL: Fluorescence
PA: Photoacoustic
EPR: Enhanced permeability and retention
