# Correction: Multifunctional nanobubbles carrying indocyanine green and paclitaxel for molecular imaging and the treatment of prostate cancer

**DOI:** 10.1186/s12951-025-03174-8

**Published:** 2025-02-06

**Authors:** Minmin Lan, Lianhua Zhu, Yixuan Wang, Daijia Shen, Kejing Fang, Yu Liu, Yanli Peng, Bin Qiao, Yanli Guo

**Affiliations:** 1 Department of Ultrasound, Southwest Hospital, Army Medical University, No. 30 Gaotanyan Street, Shapingba District, Chongqing, 400038 China; 2 State Key Laboratory Of Silkworm Genome Biology, Southwest University, Beibei District, Chongqing, China; 3 Chongqing Medical University, Chongqing, China

**Journal of Nanobiotechnology (2020) 18:121**

10.1186/s12951-020-00650-1

Following publication of the original article, the authors identified an error in PTX group (TUNEL-stained image), in Fig. 8. The incorrect and revised versions of the figure are shown in this Correction and the original article has been corrected. All authors sincerely apologize for this error.

Incorrect Fig. 8.

**Fig. 8:**
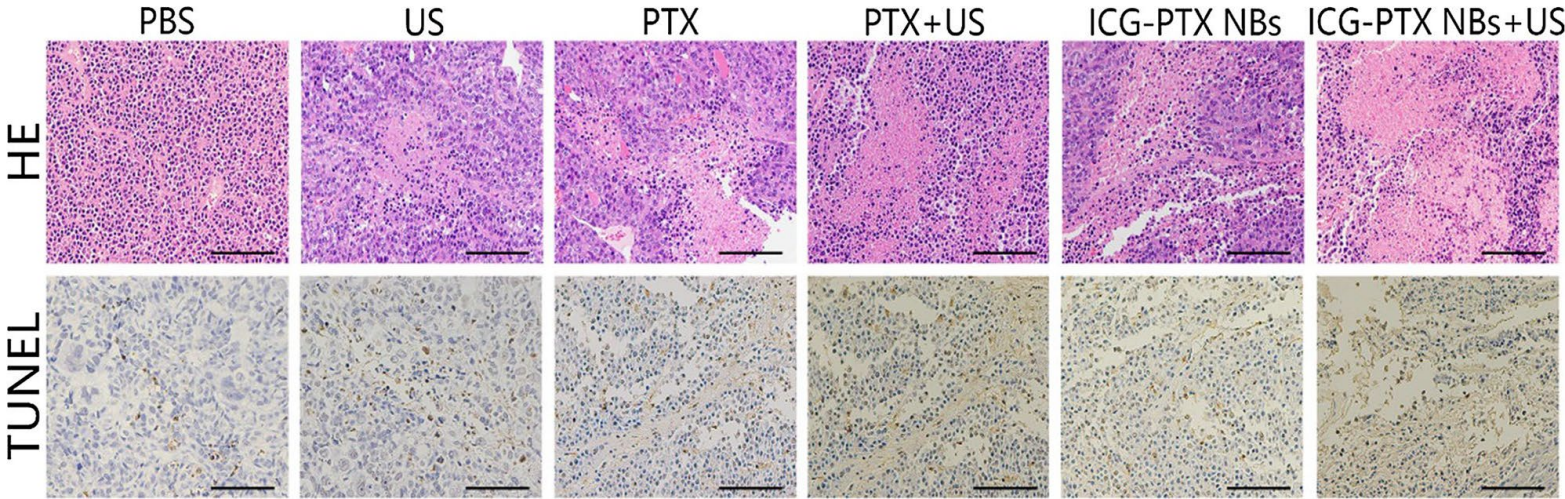
H&E- and TUNEL-stained images of tumour tissue sections from xenograft-bearing mice receiving diferent treatments after 18 days of treatment. H&E assay results revealed blue staining of the cell nuclei and red staining of the cytoplasmic and extracellular matrix. TUNEL assay analysis showed that the brown staining of the cell nuclei indicated apoptosis- and proliferation-positive tumour cells, whereas blue staining of the cell nuclei indicated apoptosis- and proliferation-negative tumour cells. Scale, 100 μm

Correct Fig. 8.

**Fig. 8:**
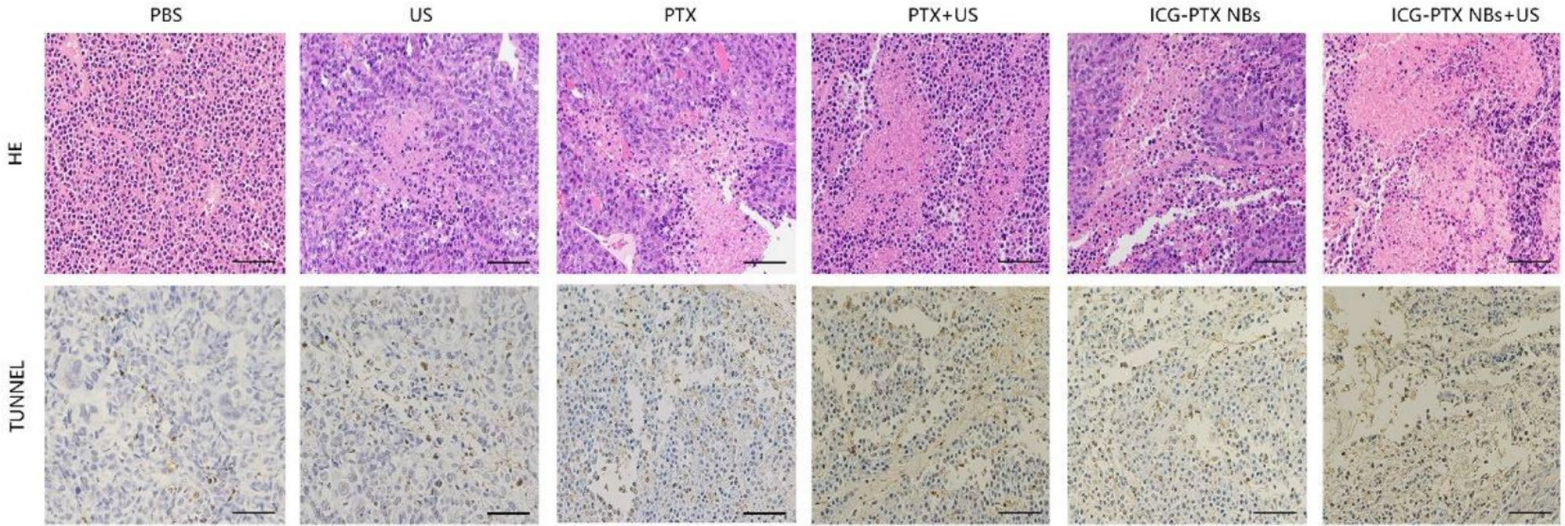
H&E- and TUNEL-stained images of tumour tissue sections from xenograft-bearing mice receiving diferent treatments after 18 days of treatment. H&E assay results revealed blue staining of the cell nuclei and red staining of the cytoplasmic and extracellular matrix. TUNEL assay analysis showed that the brown staining of the cell nuclei indicated apoptosis- and proliferation-positive tumour cells, whereas blue staining of the cell nuclei indicated ssapoptosis- and proliferation-negative tumour cells. Scale, 100 μm

